# Acquired-resistance of bevacizumab treatment for radiation brain necrosis: a case report

**DOI:** 10.18632/oncotarget.7724

**Published:** 2016-02-25

**Authors:** Hongqing Zhuang, Xiangkun Yuan, Dayong Sun, Jianliang Bian, Joe Y. Chang, Zhiyong Yuan, Ping Wang

**Affiliations:** ^1^ Department of Radiotherapy, Tianjin Medical University Cancer Institute and Hospital, National Clinical Research Center for Cancer, Tianjin Key Laboratory of Cancer Prevention and Therapy, Tianjin, China; ^2^ Department of Radiotherapy, Hebei Province Cangzhou Hospital of Integrated Traditional and Western Medicine, Hebei, China; ^3^ Department of Chemo-Radiotherapy, Chengde Center Hospital, Second Clinical Medical School of Chengde Medical University, Hebei, China; ^4^ Department of Radiotherapy, Affiliated Hospital of Hebei University, Hebei, China; ^5^ Department of Radiation Oncology, Division of Radiation Oncology, The University of Texas, MD Anderson Cancer Center, Houston, TX, USA

**Keywords:** bevacizumab, acquired resistance, radiation brain necrosis

## Abstract

The case study reported on acquired bevacizumab resistance in one patient receiving re-treatment with bevacizumab following radiation brain necrosis progression after bevacizumab was discontinued. This case offers novel and additional insight for bevacizumab treatment. Low-dose bevacizumab is effective for radiation brain necrosis, and radiation brain necrosis may progress after bevacizumab discontinuation, whereas too many cycles of bevacizumab treatment may induce drug-resistance and re-treatment failure following the progression. Therefore, more rational administration for radiation brain necrosis with bevacizumab may include three aspects: short-course treatment, timely discontinuation upon obtaining satisfactory effects (to prevent long-term medication associated resistance) and re-treatment after brain necrosis progression.

Efficacy of bevacizumab in treating radiation brain necrosis has been reported in many studies [1-8], however, hardly any data about acquired bevacizumab resistance in patients re-treated with bevacizumab following radiation brain necrosis progression after the drug discontinued is presented. In this paper, we report one case of such acquired resistance following treatment with bevacizumab for radiation brain necrosis.

A 56-year-old female patient was admitted to our hospital in August 2011 with a complaint of cough and hemoptysis for 6 months, and dizziness for 1 month. She received a diagnosis of left lung adenocarcinoma with metastasis in the mediastinal lymph nodes and left parietal lobe at clinical stage IV, T1N2M1. Genetic testing showed mutations in the EGFR19 exons and she was prescribed oral erlotinib. In February 2012, she exhibited symptoms including dizziness and nausea, and underwent CyberKnife stereotactic radiotherapy of the left parietal metastasis. The gross tumor volume (GTV) was determined using enhanced computed tomography (CT), and the planning target volume (PTV) considered an area of 1.5 mm from GTV. Furthermore, 85% of the radiation dose (total dose 36Gy/3f) targeted the PTV. A follow-up in April 2012 showed that the intracranial metastasis was smaller with a symptom improvement. In September 2012, however, she developed symptoms such as headache and dizziness again, and a follow-up with enhanced-magnetic resonance imaging (MRI) showed a significantly larger lesion represented with map-like enhancement and a large edema area in the surrounding tissue. She was further diagnosed with radiation brain necrosis based on the spectroscopy. From September 2012 to July 2013, she received 10 cycles of bevacizumab treatment (5 mg/kg, q3w-4w, she weighed about 60 kg and we gave her 300mg once). During the treatment, MRI and MRS tests showed that the brain necrosis was significantly improved after she took bevacizumab. Her symptoms disappeared, and bevacizumab was discontinued. In June 2014, she exhibited headache and dizziness again. Brain MRI showed significant progression of necrosis lesion compared with the results in July 2013, and spectroscopy indicated brain necrosis further. From August 2014 to March 2015, she received a total of 7 cycles of bevacizumab treatment (using the same administration as before; she did not take bevacizumab on June 10, 2014 because of persistent bleeding and delayed healing after fingernail extraction due to severe tarceva-induced paronychia). However, the cerebral edema-related symptoms did not significantly improve at that time; and bevacizumab was discontinued in March 2015. A brain MRI performed in March 2015 showed no significant improvement in brain necrosis from June 2014, and spectroscopy still indicated radiation brain necrosis.

**Figure 1:**
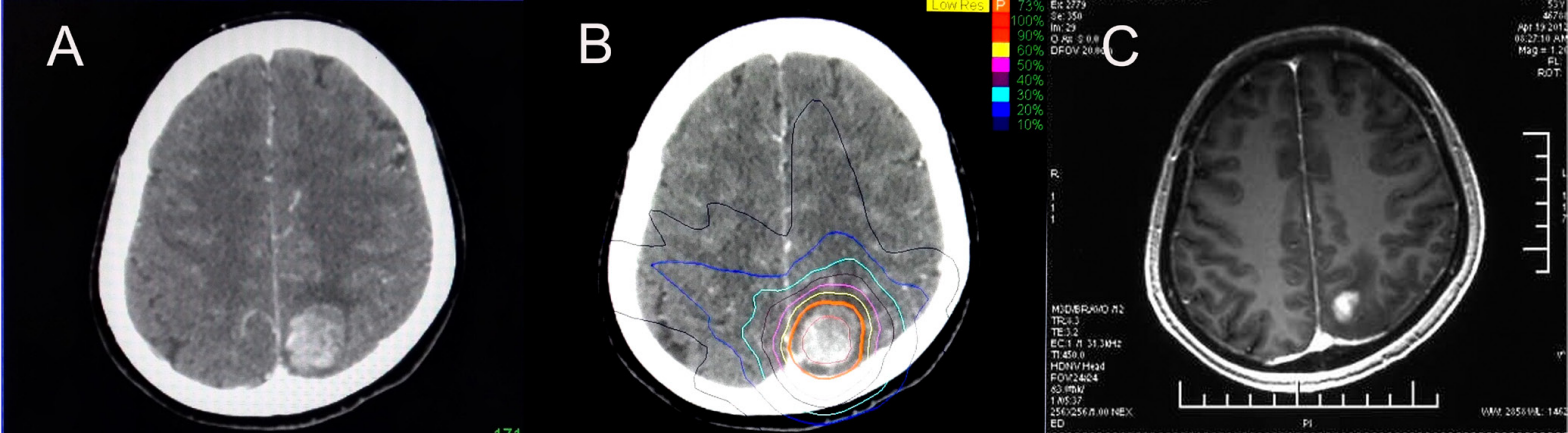
CyberKnife treatment and effects **A.** CT scan before treatment (February 2012); **B.** a follow-up in April 2012 showed a significantly smaller lesion.

**Figure 2:**
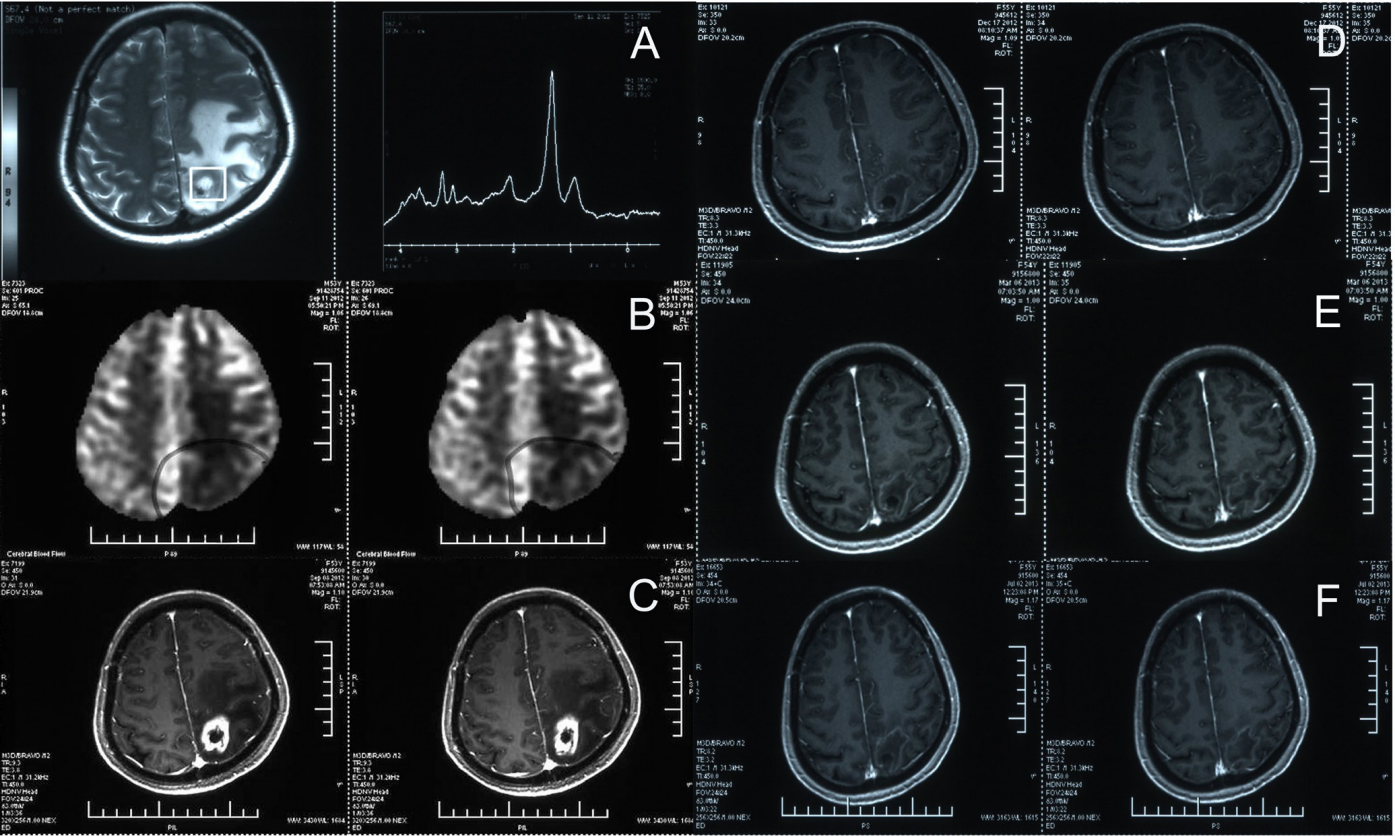
Management of brain necrosis during bevacizumab treatment **A.** In September 2012, magnetic resonance spectroscopy (MRS) showed a significant increase in lipid peaks (LL: 108964, Cho:33671, Cr:29534, NAA:31278). **B.** In September 2012, magnetic resonance perfusion imaging showed significantly less perfusion at the original treatment site. **C.** In September 2012, enhanced brain MRI showed map-like enhancement and a large edema in the surrounding tissue. Based on the three results (A, B and C), the patient was diagnosed with radiation brain necrosis, and received 10 cycles of bevacizumab at 300 mg, q3-4w* from September 2012 to July 2013. **D.** A follow-up in December 2012 (after 3 cycles of bevacizumab treatment) showed a significant improvement in brain necrosis. **E.** A follow-up in March 2013 (after 6 cycles of bevacizumab treatment); **F.** a follow-up in July 2013 (after 10 cycles of bevacizumab treatment). As indicated, during the bevacizumab treatment, multiple follow-up visits showed a significant improvement in brain necrosis after the bevacizumab treatment applied, and the lesion remained relatively stable.

## DISCUSSION

Many previous studies have shown that development of brain necrosis is closely related to vascular injury and angiogenesis [9-11]. As an anti-angiogenesis drug, bevacizumab can prune vessels and regulate vascular permeability, and studies have demonstrated that it relieves brain necrosis associated cerebral hydropic symptoms. The current case also validates the efficacy of bevacizumab treatments, and offers additionally novel insight further. Firstly, the patient received bevacizumab at 300mg, q3-4w, with good results, which shows that low-dose bevacizumab can produce satisfactory short-term outcome for radiation brain necrosis. Secondly, radiation brain necrosis may progress following bevacizumab discontinuation, which is consistent with the previous consensus that necrosis is irreversible in theory once formed. Besides, the patient received a relatively more cycles of bevacizumab treatment (10 cycles) during the initial therapy. It is believed that the patient was likely to develop acquired bevacizumab resistance which contributed to re-treatment failure following the cerebral necrosis progression, although some other researchers believed that bevacizumab resistance is caused by hypoxia due to bevacizumab over-pruning new vessels, however the over-pruning theory was only supported by few literatures and not mature and convincing enough, making the excessive pruning is only one potential mechanism [12-14]. Moreover, bevacizumab regimen also needs further consideration. It is more rational that bevacizumab is given in short-course, stopped timely upon obtaining satisfactory effects (to prevent long-term medication associated resistance) and re-prescribed after brain necrosis progression.

**Figure 3:**
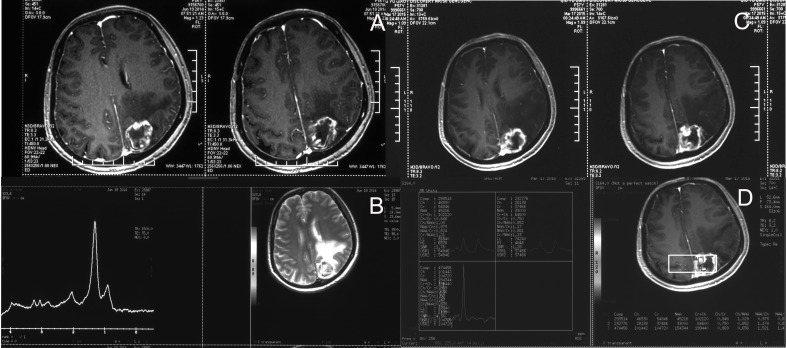
Brain necrosis progression and ineffective bevacizumab re-treatment **A.** In June 2014, enhanced MRI showed progression of the radiation brain necrosis; **B.** In June 2014, MRS indicated radiation brain necrosis(LL: 114334, Cho:20567, Cr:20432, NAA:38623), and the patient received 7 cycles of bevacizumab re-treatment from August 2014 to March 2015. **C.** In March 2015, enhanced MRI showed a more pronounced enhancement of the lesion than before and indicated treatment for brain necrosis was ineffective. **D.** In March 2015, MRS indicated brain necrosis (Cho:28138, Cr:37466, NAA:33000).
